# Comprehensive molecular diagnosis of 67 Chinese Usher syndrome probands: high rate of ethnicity specific mutations in Chinese USH patients

**DOI:** 10.1186/s13023-015-0329-3

**Published:** 2015-09-04

**Authors:** Lichun Jiang, Xiaofang Liang, Yumei Li, Jing Wang, Jacques Eric Zaneveld, Hui Wang, Shan Xu, Keqing Wang, Binbin Wang, Rui Chen, Ruifang Sui

**Affiliations:** Department of Ophthalmology, Peking Union Medical College Hospital, Peking Union Medical College, Chinese Academy of Medical Sciences, 1 Shuai Fu Yuan, Beijing, 10073 China; Human Genome Sequencing Center, Baylor College of Medicine, Houston, TX USA; Department of Molecular and Human Genetics, Baylor College of Medicine, Houston, TX 77030 USA; Structural and Computational Biology and Molecular Biophysics Program, Baylor College of Medicine, Houston, TX 77030 USA; Program in Developmental Biology, Baylor College of Medicine, Houston, TX 77030 USA; Department of Medical Genetics, School of Basic Medical Sciences, Capital Medical University, Beijing, China; Graduate School of Peking Union Medical College, Beijing, China; National Research Institute for Family Planning, Beijing, China

## Abstract

**Background:**

Usher syndrome (USH) is the most common disease causing combined deafness and blindness. It is predominantly an autosomal recessive genetic disorder with occasionally digenic cases. Molecular diagnosis of USH patients is important for disease management. Few studies have tried to find the genetic cause of USH in Chinese patients. This study was designed to determine the mutation spectrum of Chinese USH patients.

**Methods:**

We applied next generation sequencing to characterize the mutation spectrum in 67 independent Chinese families with at least one member diagnosed with USH. Blood was collected at Peking Union Medical College Hospital. This cohort is one of the largest USH cohorts reported. We utilized customized panel and whole exome sequencing, variant analysis, Sanger validation and segregation tests to find disease causing mutations in these families.

**Results:**

We identified biallelic disease causing mutations in known USH genes in 70 % (49) of our patients. As has been previously reported, *MYO7A* is the most frequently mutated gene in our USH type I patients while *USH2A* is the most mutated gene in our USH type II patients. In addition, we identify mutations in *CLRN1*, *DFNB31*, *GPR98* and *PCDH15* for the first time in Chinese USH patients. Together, mutations in *CLRN1, DNFB31, GPR98* and *PCDH15* account for 11.4 % of disease in our cohort. Interestingly, although the spectrum of disease genes is quite similar between our Chinese patient cohort and other patient cohorts from different (and primarily Caucasian) ethnic backgrounds, the mutations themselves are dramatically different. In particular, 76 % (52/68) of alleles found in this study have never been previously reported. Interestingly, we observed a strong enrichment for severe protein truncating mutations expected to have severe functional consequence on the protein in USH II patients compared to the reported mutation spectrum in RP patients, who often carry partial protein truncating mutations.

**Conclusions:**

Our study provides the first comprehensive genetic characterization of a large collection of Chinese USH patients. Up to 90 % of USH patients have disease caused by mutations in known USH disease genes. By combining NGS-based molecular diagnosis and patient clinical information, a more accurate diagnosis, prognosis and personalized treatment of USH patients can be achieved.

**Electronic supplementary material:**

The online version of this article (doi:10.1186/s13023-015-0329-3) contains supplementary material, which is available to authorized users.

## Background

Usher Syndrome (USH) is the most common disease of combined deafness and blindness. It is characterized by sensorineural hearing loss (SNHL), retinitis pigmentosa (RP), and manifests with or without vestibular dysfunction. Depending on the age of onset, the severity of the retinal and hearing phenotypes and the presence or absence of vestibular dysfunction, USH is classified into three major categories. USH I is characterized by congenital deafness with severe balance problems. USH I patients develop vision problems in early childhood. USH II patients are born with moderate to severe hearing loss, normal balance, and usually develop RP during adolescence. USH III patients have normal or near normal balance, progressive hearing loss, and vision problems varying in severity which usually develop during adolescence [1]. Genetically, USH is genetically heterogeneous, with 12 known disease genes and 3 additional loci having been identified so far [1–4]. USH is predominantly a recessive disease, though other inheritance patterns occur at lower frequency. For instance, a digenic USH can be caused by simultaneous mutations in both PCDH15 and CDH23 [1]. In addition, truncation of PDZD7 has been reported as a modifier of GPR98 and USH2A mutations [5]. It is also worth noting that mutations in many of the USH genes can also lead to deafness without a retinal phenotype [6–9].

Given the high clinical and genetic complexity underlying USH, molecular screening for mutations in USH genes significantly improves diagnosis. Next generation sequencing (NGS) is emerging as a cost-efficient technology for sequencing a large number of genes [10, 11]. NGS is ideally suited for molecular diagnosis of USH for two reasons. First, many USH disease genes have many isoforms with a large number of exons. In total, more than 400 coding exons have been annotated in known USH genes. As a result, mutation screening for all coding exons by Sanger sequencing is cost prohibitive while NGS is feasible. Second, a large diversity of pathogenic alleles of various types has been reported and novel mutations are frequent, making array based diagnosis inaccurate. Indeed, in a recent report a European USH patient cohort was screened for mutations by Sanger sequencing. Interestingly, 48 % of the alleles identified were novel [12]. The rate of novel mutations is expected to be even higher in poorly studied populations like our Chinese cohort. Therefore, a sequence-based approach is necessary to achieve high diagnosis rate.

Although USH patients of European descent have been under intense investigation [1], only a small number of studies have been published on Chinese USH cohorts, each with a no more than 10 cases [13–17]. Mutations in Chinese USH patients from these studies occurred only in two genes, *MYO7A* and *USH2A*. Furthermore, founder mutations specific for many ethnic groups have been identified. For example, the founder mutation c.8559-2A > G in*USH2A* accounts for 26 % of all Western Japanese USH patients but was never observed in Europeans. Similarly, the most prevalent mutation in European populations, c.2299delG in *USH2A*, has never been observed in Asian patients [18, 19]. To gain insight into the molecular basis of Chinese USH patients we performed comprehensive NGS of all known USH genes in a cohort of 67 probands and their families. Indeed, our cohort has a different mutation spectrum than that of patients of European descent.

## Methods

### Clinical diagnosis of USH and sample collection

All subjects were initially enrolled at Peking Union Medical College Hospital (PUMCH). During their initial visit, a full medical and family history was recorded, pedigrees were drawn, and ophthalmological examinations were performed. Each patient underwent a standard ophthalmic examination including best correct visual acuity (BCVA) according to projected Snellen charts, slit-lamp biomicroscopy, dilated indirect ophthalmoscopy, fundus photography if possible, and visual field tests (Octopus,Interzeag, Schlieren, Switzerland). Retinal structure was examined by optical coherence tomography (OCT) (Topcon, Tokyo, Japan). Electroretinograms (ERGs) were performed (RetiPort ERG system, Roland Consult, Wiesbaden, Germany) using corneal “ERGjet” contact lens electrodes. The ERG protocol complied with the standards published by the International Society for Clinical Electrophysiology of Vision. Auditory examinations including otoscopic exploration, pure-tone and speech audiometry were conducted by Otolaryngologists.

The diagnosis of USH was based on previously reported criteria [20]. Written informed consent was obtained from all participating individuals or their guardians. Genomic DNA was isolated from peripheral leukocytes using QIA amp DNA Blood Midi Kit (QIAGEN, Hilden, Germany) according to the manufacturer’s protocol. This study was approved by the Institutional Review Board of PUMCH and adhered to the tenets of the Declaration of Helsinki and the Guidance on Sample Collection of Human Genetic Diseases by the Ministry of Public Health of China.

### Design of retinal disease capture panel

A capture panel of retinal disease genes was developed and assessed by our group [10, 11]. All annotated coding exons and flanking splicing sites for 9 USH genes *(MYO7A, PCDH15, CDH23, USH1C, USH1G, USH2A, GPR98, DNFB31, and CLRN1)* and one USH modifier gene *PDZD7* were included in the capture design. In total, the panel included 196 known retinal disease genes (Additional file 1: Table S1). For all patients without a positive molecular diagnosis, mutations in other recently reported USH disease genes, *CIB2*, *HARS* and *ABHD12*, were screened by whole exome sequencing. Whole exome sequencing was performed as described previously [21].

### Panel capture sequencing

About 50 pre-capture libraries were pooled together for one panel capture reaction. Agilent Hybridization and Wash Kits were used for panel capture, following the standard manufacturer’s protocol. Captured libraries were sequenced on the Illumina HiSeq 2000 as 100-bp paired-end reads, following the manufacturer's protocols. Whole exome sequencing library construction, capture and sequencing was performed as previously described [21].

### Bioinformatics analysis of sequencing results and pathogenic mutation identification

Sequence data was processed through an automated pipeline developed in house as previously described [10, 11]. Briefly, raw reads were mapped to the hg19 reference genome followed by variant calling including SNPs and indels. Variants were then filtered against both publicly available databases and internal databases with a cut off frequency of less than 0.5 % in the general population. The HGMD professional database (http://www.biobase-international.com/product/hgmd) and USH bases (https://grenada.lumc.nl/LOVD2/Usher_montpellier/USHbases.html) [22] were used to search for known pathogenic mutations. We utilized a previously described stepwise strategy to systematically identify the putative pathogenic mutations for each USH family. Mutations in 9 known USH genes were checked for, in order, known pathogenic mutations, novel loss-of-function mutations, and novel missense mutations. In cases where missense mutations segregated with disease, they were considered as pathogenic even if their functional predictions were neutral. We also considered reported digenic inheritance of *PCDH15*/*CDH23*, *PDZD7*/*GPR98* and *PDZD7*/*USH2A*. We only considered monoallelic mutations if they were reported pathogenic missense mutations, nonsense mutations, frameshift mutation and splice site mutations in known USH genes. The same prioritizing strategy was applied to other retinal disease genes and we only picked mutations that fit the disease model of a gene. Sanger validation was performed for all putative causal pathogenic mutations. Segregation tests were performed when additional family members were available.

## Results

### Recruitment of 67 USH families and clinical diagnosis

**Fig. 1:**
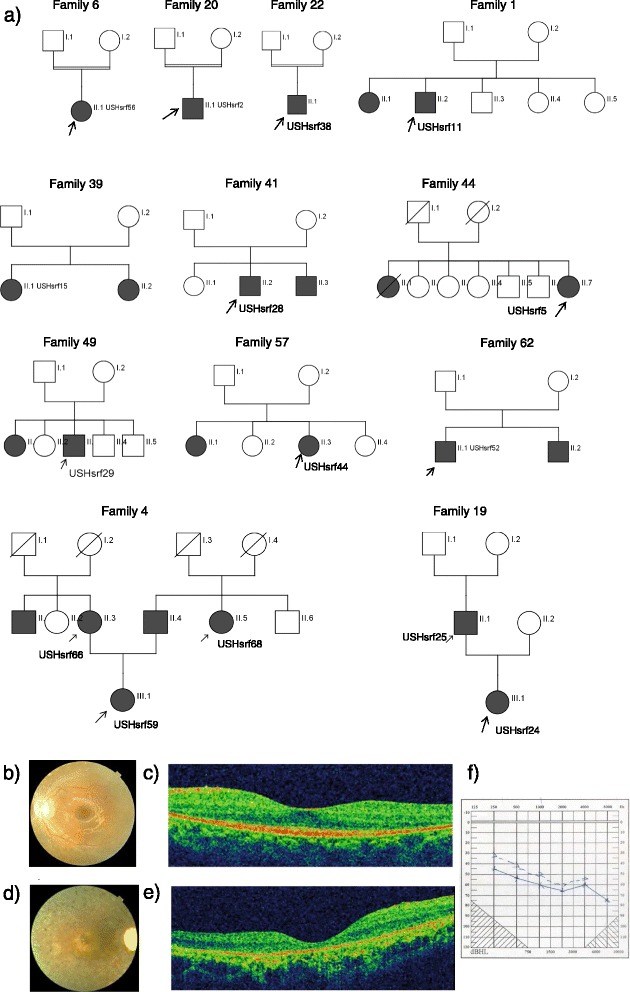
Pedigrees of non-simplex and consanguineous families and sample example figures of clinical data. **a** Pedigrees of non-simplex and consanguineous families. USH patients are illustrated by squares or circles in black while the unaffected family members are in white. Patients with DNA sequenced by panel or whole exome sequencing in our project are indicated by an arrow. **b** Fundus of left eye of USHsrf59 at age 31. The fundus showed salt and pepper pigmentation variation in the periphery retina and attenuation of the retinal vessels. **c** OCT of left eye of USHsrf59 at age 31. OCT showed lack of IS/OS except macula fovea in photoreceptor layer. Her visual acuity is 0.8/0.5 at age 31. This patient was diagnosed with USH II. Her hearing loss began at age 5 and vision loss began at age 12. **d** Fundus of right eye of USHsrf66 at age 57. The fundus showed bone spicule pigmentation variation and attenuation of the retinal vessels. **e** OCT of left eye of USHsrf66 at age 57. Her visual acuity is 0.06/0.06 at age 57. OCT showed a thinned retinal pigment epithelium and a photoreceptor layer (lack of IS/OS). This patient was diagnosed with USH II. Her hearing loss began at age 8 and vision loss began at age 30 with night blindness starting from school age. **f** Hearing test on left ear of USHsrf66

**Fig. 2:**
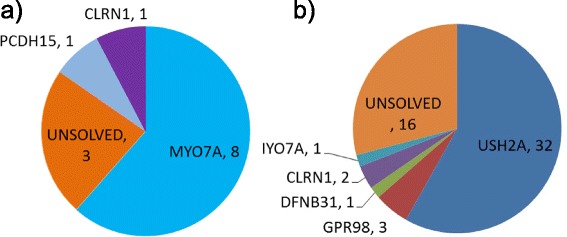
Another sample figure title Summary of mutations identified in USH genes. **a** Genes mutated in USH I patients. **b** Genes mutated in USH II patients

In this study, we recruited a total of 70 patients from 67 unrelated USH families from different regions of China. This group contained 14 patients diagnosed with USH type I, 54 patients as USH type II or USH type II-like, 1 patient as USH type III, and 1 patient with an undetermined subtype. In most families, the proband was the only affected member in the family, including three patients from consanguineous marriages (USHsrf2, USHsrf38, and USHsrf56) (Fig. 1). Two families, USHsrf24 and USHsrf66, have multiple affected members. In family USbHsrf24, both the father and the daughter were diagnosed with USH II. As shown in Fig. 1, the USHsrf66 family is a large family with five affected members, including USHsrf66, USHsrf68, and USHsrf59 who were recruited for this study. Detailed clinical information pertaining to these families is included in Additional file 1: Table S3. All our patients exhibited phenotypes consistent with USH syndrome [20]. All patients’ clinical phenotypes are listed in Additional file 1: Table S3, while representative fundus images and hearing test results are shown in Fig. 2.

### Mutation screening for known USH and other eye disease genes

A gene capture panel containing196 known retinal disease genes was developed in our lab and has effectively identified mutations in known retinal disease genes [10, 11]. We applied this retinal disease gene panel to our USH patients and excellent coverage was achieved with an average coverage of 109X for target genes (Additional file 1: Table S2). On average, 96.8 % of the target region was sequenced with more than 10X coverage which is sufficient to call heterozygous mutations.

An in-house automatic variant calling, filtering, and annotation pipeline was used to analyse the sequencing data. Filtering against multiple public and internal databases, only rare SNPS and indels (defined as a frequency < 0.5 % in 20,000 controls) were retained for each patient. Each variant was further annotated and the ones that do not affect protein coding were further removed. As a result, on average 15 rare variants in all known retinal disease genes, including 3 in the USH disease genes, were identified per patient. The pathogenicity of these variants was further evaluated as described in the materials and methods section.

### Mutations were identified in 10 out of 14 USH I patients

Biallelic mutations in known USH genes were detected in 10 USH I patients (Fig. 2a). Consistent with previous reports, *MYO7A* was the most frequently mutated gene with 13 different pathogenic mutations found in 8 patients (Table 1). Among them, 3 have been previously reported as pathogenic alleles while the other 10 alleles are novel, including 3 frameshift mutations, 2 splicing site mutations, 3 nonsense mutations, and 2 missense mutations (Table 1). Both novel missense alleles, *MYO7A*: c. 2837 T > G: M946R and *MYO7A*: c. 5396 T > C: P.L1799P, are likely to be pathogenic based on the following evidence. First, both are extremely rare in the control population and have not been observed in any of the public or our internal variant databases that together contain around 20,000 individuals. Second, the amino acids M946 and L1799 are highly conserved across vertebrates and all the way to invertebrates (Additional file 2: Figure S1). Third, *in silico* prediction of M946R and L1799P variants suggests that they are likely to be detrimental (Additional file 1: Table S6). Finally, both variants segregated with disease within the families in which they occurred.

**Table 1:** Biallelic mutations in USH genes in USH I patients^a^

Patient	Gene	Type	NMID	Exon	cDNA	Amino acid	Genotype	Patient origin	Reference
USHsrf17	MYO7A	Splicing	NM_001127180	19	c2187 + 1G > A^b^	c2187 + 1G > A	Heterozygous	Chinese	Novel
	MYO7A	frameshift	NM_001127180	5	c.390_391insC^c^	p.M130fs	Heterozygous	Chinese	Novel
USHsrf22	MYO7A	nonsynonymous	NM_001127180	17	c.C1969T	p.R657W	Heterozygous	UK	[37]
	MYO7A	nonsynonymous	NM_001127180	7	c.C721T	p.R241C	Heterozygous	England, Scoland	[38]
USHsrf41	MYO7A	frameshift	NM_001127180	29	c.3695_3705del	p.1232_1235del	Heterozygous	Chinese	Novel
	MYO7A	stopgain	NM_001127180	33	c.G4398A	p.W1466X	Heterozygous	Chinese	Novel
USHsrf44	MYO7A	nonframeshift	NM_001127180	43	c.5766_5768del	p.1922_1923del	Heterozygous	ALL types	pathogenic in dbSNP
	MYO7A	stopgain	NM_001127180	40	c.C5467T	p.R1823X	Heterozygous	Chinese	Novel
USHsrf53	MYO7A	stopgain	NM_001127180	33	c.C4354T	p.Q1452X	Heterozygous	Chinese	Novel
	MYO7A	nonsynonymous	NM_001127180	23	c.T2837G	p.M946R	Heterozygous	Chinese	Novel
USHsrf56	MYO7A	nonframeshift	NM_001127180	43	c.5766_5768del^d^	p.1922_1923del	Homozygous	ALL types	pathogenic in dbSNP
USHsrf61	MYO7A	frameshift	NM_001127180	29	c.3695_3705del	p.1232_1235del	Heterozygous	Chinese	Novel
	MYO7A	frameshift	NM_001127180	32	c.4251delC	p.I1417fs	Heterozygous	Chinese	Novel
USHsrf39	MYO7A	Splicing	NM_001127180	32	c.4323 + 2 T > C	c.4323 + 2 T > C	Heterozygous	Chinese	Novel
	MYO7A	nonsynonymous	NM_001127180	40	c.T5396C	p.L1799P	Heterozygous	Chinese	Novel
USHsrf8	PCDH15	frameshift	NM_001142773	14	c.1799_1800insTA	p.S600fs	Heterozygous	Chinese	Novel
	PCDH15	stopgain	NM_001142773	21	c.A2893T	p.R965X	Heterozygous	Chinese	Novel
USHsrf14	CLRN1	stopgain	NM_001195794	4	c.C658T	p.R220X	Heterozygous	Chinese	Novel
	CLRN1	nonsynonymous	NM_001195794	1	c.G190A	p.G64R	Heterozygous	Chinese	Novel

^a^Unless stated otherwise, alleles are not found in any of the database we used for control common variants

^b^rs111033280;CLN;PM;LSD

^c^0.000227 in ESP6500

^d^1/2184 in 1000 genome

Additional putatively pathogenic mutations were identified in *PCHD15* and *CLRN1* in this group of USH I patients. Patient USHsrf8 was found to carry compound heterozygous mutations in *PCDH15*, consisting of the novel frameshift mutation *PCDH15*:c.1799_1800insTA:p.S600fs and the novel nonsense mutation *PCDH15*:c. 2893A > T: p.R965X (Table 1). Interestingly, patient USHsrf14 had an unexpected molecular diagnosis because patient USHsrf14 was diagnosed with USH I but has mutations in *CLRN1* which have previously been reported to cause mostly USH III [23]. In one previous case, a patient with USH I was reported to have a *CLRN1* frameshift mutation [24]. Together with our study, this may indicate certain *CLRN1* mutations can cause USH I. This patient had severely impaired hearing at a very young age and got cochlear implants around age of 6. She experienced poor night vision and wore glasses before she turned 10. This patient was found to carry novel nonsense mutation *CLRN1*:c. 658C > T:p. R220X and novel missense mutation *CLRN1*:c. 190G > A:pG64R, which is predicted to be pathogenic (Additional file 1: Table S6). The nonsense mutation is from the patient’s father while the missense mutation is from the patient’s mother, and the mutation thus segregated with disease in the small pedigree.

### Mutations were identified in 39 of 54 USH II and atypical patients

Biallelic mutations were detected in 39 USH II or USH II-like patients (Table 2), with *USH2A* mutated in 32 patients, *GRP98* mutated in 3 patients, *CLRN1* mutated in 2 patients, *MYO7A* mutated in 1 patient, and *DFNB31* mutated in 1 patient (Fig. 2b).

**Table 2:** Biallelic mutations in USH genes in USH II patients^a^

Patient	Gene	Type	NMID	Exon	cDNA	Amino acid	Genotype	Patient origin	Reference
USHsrf1	USH2A	Splicing	NM_206933	44	c.8559-2A > G	c.8559-2A > G	Heterozygous	Japanese	[31]
	USH2A	nonsynonymous	NM_206933	5	c.G802A^d^	p.G268R	Heterozygous	uk	[39]
USHsrf2	USH2A	stopgain	NM_206933	49	c.C9723A	p.Y3241X	Homozygous	Chinese	Novel
USHsrf7	USH2A	stopgain	NM_206933	54	c.C10612T	p.R3538X	Heterozygous	Chinese	Novel
	USH2A	stopgain	NM_206933	68	c.C14911T	p.R4971X	Heterozygous	UK	[30]
USHsrf9	USH2A	Splicing	NM_206933	44	c.8559-2A > G	c.8559-2A > G	Heterozygous	Japanese	[31]
	USH2A	nonsynonymous	NM_206933	28	c.G5581A^c^	p.G1861S	Heterozygous	Chinese	Novel
USHsrf10	USH2A	frameshift	NM_206933	9	c.1589_1590insG	p.T530fs	Heterozygous	Chinese	Novel
	USH2A	frameshift	NM_206933	28	c.5735_5736del	p.1912_1912del	Heterozygous	Chinese	Novel
USHsrf11	USH2A	frameshift	NM_206933	2	c.100_101insT^e^	p.R34fs	Heterozygous	Denmark and Norway?	[40]
	USH2A	Splicing	NM_206933	44	c.8559-2A > G	c.8559-2A > G	Heterozygous	Japanese	[31]
USHsrf18	USH2A	frameshift	NM_206933	2	c.100_101insT^e^	p.R34fs	Heterozygous	Denmark and Norway?	[40]
	USH2A	nonsynonymous	NM_206933	42	c.G8232C	p.W2744C	Heterozygous	Chinese	[15]
USHsrf20	USH2A	Splicing	NM_206933	44	c.8559-2A > G	c.8559-2A > G	Heterozygous	Japanese	[31]
	USH2A	nonsynonymous	NM_206933	5	c.G802A^d^	p.G268R	Heterozygous	UK	[39]
USHsrf21	USH2A	frameshift	NM_206933	17	c.3420_3423del	p.1140_1141del	Heterozygous	Chinese	Novel
	USH2A	stopgain	NM_206933	47	c.G9319T	p.E3107X	Heterozygous	Chinese	Novel
USHsrf23	USH2A	frameshift	NM_206933	63	c.13060_13063del	p.4354_4355fsdel	Heterozygous	Chinese	Novel
	USH2A	frameshift	NM_206933	67	c.14667delG	p.G4889fs	Heterozygous	Chinese	Novel
USHsrf24	USH2A	Splicing	NM_206933	8	c.1144-2A > C	c.1144-2A > C	Heterozygous	Chinese	Novel
	USH2A	stopgain	NM_206933	35	c.C6752A	p.S2251X	Heterozygous	Chinese	Novel
USHsrf25	USH2A	stopgain	NM_206933	35	c.C6752A	p.S2251X	Heterozygous	Chinese	Novel
	USH2A	nonsynonymous	NM_206933	50	c.C9815T	p.P3272L	Heterozygous	Dutch	[41]
USHsrf30	USH2A	frameshift	NM_206933	63	c.12409delA	p.R4137fs	Heterozygous	Chinese	Novel
	USH2A	nonsynonymous	NM_206933	13	c.T2802G^b^	p.C934W	Heterozygous	Chinese	[15]
USHsrf31	USH2A	nonsynonymous	NM_206933	13	c.T2802G^b^	p.C934W	Homozygous	Chinese	[15]
USHsrf32	USH2A	stopgain	NM_206933	48	c.C9469T	p.Q3157X	Heterozygous	Japanese	[18]
	USH2A	nonsynonymous	NM_206933	14	c.T2914G	p.C972G	Heterozygous	Chinese	Novel
USHsrf33	USH2A	Splicing	NM_206933	10	c.1644 + 1G > A	c.1644 + 1G > A	Heterozygous	Chinese	Novel
	USH2A	stopgain	NM_206933	12	c.1993_1994insT	p.K665_R666delinsX	Heterozygous	Chinese	Novel
USHsrf35	USH2A	stopgain	NM_206933	34	c.G6488A	p.W2163X	Heterozygous	Chinese	Novel
	USH2A	nonsynonymous	NM_206933	50	c.G9958T	p.G3320C	Heterozygous	Chinese	Novel
USHsrf36	USH2A	nonsynonymous	NM_206933	6	c.C1000T	p.R334W	Homozygous	all types	[40]
USHsrf37	USH2A	nonsynonymous	NM_206933	40	c.A7492T	p.S2498C	Heterozygous	Chinese	Novel
	USH2A	nonsynonymous	NM_206933	6	c.G1048A^f^	p.V350I	Heterozygous	Chinese	Novel
USHsrf45	USH2A	Splicing	NM_206933	44	c.8559-2A > G	c.8559-2A > G	Heterozygous	Japanese	[31]
	USH2A	stopgain	NM_206933	11	c.C1876T	p.R626X	Heterozygous	French Canadian?	[42]
USHsrf46	USH2A	frameshift	NM_206933	2	c.100_101insT^e^	p.R34fs	Homozygous	Denmark and Norway?	[40]
USHsrf48	USH2A	Splicing	NM_206933	44	c.8559-2A > G	NA	Heterozygous	Japanese	[40]
	USH2A	nonsynonymous	NM_206933	26	c.G5200C	p.G1734R	Heterozygous	Chinese	[43]
USHsrf50	USH2A	Splicing	NM_206933	44	c.8559-2A > G	NA	Heterozygous	Japanese	[16]
	USH2A	stopgain	NM_206933	6	c.T1140A	p.Y380X	Heterozygous	Chinese	Novel
USHsrf52	USH2A	Splicing	NM_206933	44	c.8559-2A > G	NA	Homozygous	Japanese	[31]
	USH2A	nonsynonymous	NM_206933	2	c.G206T	p.S69I	Heterozygous	Chinese	Novel
USHsrf54	USH2A	frameshift	NM_206933	4	c.710delT	p.F237fs	Heterozygous	Chinese	Novel
	USH2A	nonsynonymous	NM_206933	13	c.T2802G	p.C934W	Heterozygous	Chinese	[15]
USHsrf55	USH2A	frameshift	NM_206933	28	c.5735_5736del	p.1912_1912del	Heterozygous	Chinese	Novel
	USH2A	nonsynonymous	NM_206933	42	c.G8232C	p.W2744C	Heterozygous	Chinese	[15]
USHsrf59	USH2A	frameshift	NM_206933	2	c.C100T	p.R34X	Heterozygous	Denmark and Norway?	[40]
	USH2A	Splicing	NM_206933	44	c.8559-2A > G	c.8559-2A > G	Heterozygous	Japanese	[31]
USHsrf60	USH2A	frameshift	NM_206933	2	c.100_101insT^e^	p.R34fs	Heterozygous	Denmark and Norway?	[40]
	USH2A	Splicing	NM_206933	44	c.8559-2A > G	c.8559-2A > G	Heterozygous	Japanese	[31]
USHsrf63	USH2A	frameshift	NM_206933	38	c.7184_7194del	p.2395_2398del	Heterozygous	Chinese	Novel
	USH2A	stopgain	NM_206933	48	c.T9453A	p.Y3151X	Heterozygous	Chinese	Novel
USHsrf66	USH2A	Splicing	NM_206933	44	c.8559-2A > G	c.8559-2A > G	Heterozygous	Japanese	[31]
	USH2A	stopgain	NM_206933	2	c.C100T	p.R34X	Heterozygous	Denmark and Norway?	[40]
USHsrf69	USH2A	stopgain	NM_206933	64	c.C13822T	p.R4608X	Heterozygous	Norway?	[39]
	USH2A	stopgain	NM_206933	15	c.G3034T	p.E1012X	Heterozygous	Chinese	Novel
USHsrf70	USH2A	frameshift	NM_206933	33	c.6347_6348insC	p.H2116fs	Heterozygous	Chinese	Novel
	USH2A	stopgain	NM_206933	41	c.A7984T	p.R2662X	Heterozygous	Chinese	Novel
USHsrf42	GPR98	frameshift	NM_032119	55	c.11547delA	p.I3849fs	Heterozygous	Chinese	Novel
	GPR98	nonsynonymous	NM_032119	32	c.G7130A	p.R2377Q	Heterozygous	Chinese	Novel
USHsrf49	GPR98	nonsynonymous	NM_032119	64	c.T13048C	p.S4350P	Heterozygous	Chinese	Novel
	GPR98	nonsynonymous	NM_032119	7	c.G929A	p.G310E	Heterozygous	Chinese	Novel
USHsrf43	GPR98	stopgain	NM_032119	32	c.C7006T	p.R2336X	Heterozygous	Chinese	Novel
	GPR98	frameshift	NM_032119	70	c.14451_14452del	p.4817_4818del	Heterozygous	Chinese	Novel
USHsrf38	DFNB31	Splicing	NM_001173425	4	c.963 + 1G > A	c.963 + 1G > A	Homozygous	Chinese	Novel
USHsrf64	CLRN1	nonsynonymous	NM_001195794	1	c.G191C	p.G64A	Homozygous	Chinese	Novel
USHsrf67	CLRN1	nonsynonymous	NM_052995	1	c.C19A	p.Q7K	Homozygous	Chinese	Novel
USHsrf40	MYO7A	nonsynonymous	NM_001127180	37	c.G4951A	p.D1651N	Heterozygous	Chinese	Novel
	MYO7A	nonsynonymous	NM_001127180	33	c.G4360A	p.V1454I	Heterozygous	Chinese	Novel

^a^Unless stated otherwise, alleles are not found in any of the database we used for control common variants

^b^1/2184 in 1000 genome

^c^0.000227 in ESP6500

^d^rs111033280;CLN;PM;LSD

^e^1/2184 in 1000 genome

^f^1/2184 in 1000 genome

**Fig. 3:**
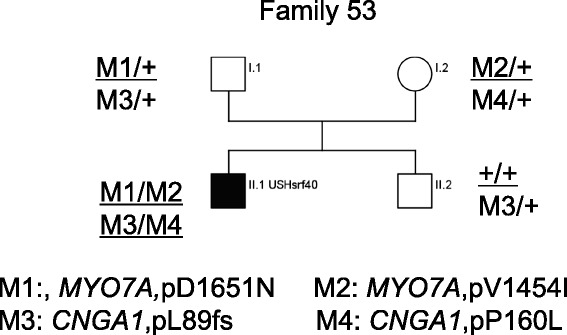
Double compound heterozygous mutations in patient USHsrf40. Patient USHsrf40 carries compound heterozygous mutations in two genes MYO7A and CGNA1: two missense mutation in MYO7A and frameshift and missense mutations in CNGA1. Mutations segregate in this family

Consistent with previous reports, we found that *USH2A* was the most frequently mutated gene in USH II patients, accounting for about 60 % (32 out of 54) of patients in this cohort. A total of 40 different mutations were identified in *USH2A,* including 27 novel alleles. The vast majority of the novel alleles (21/27) are clearly null mutations, including frameshift, splice site, and nonsense mutations (Table 2). In addition, we identified 6 novel missense mutations predicted to be pathogenic (Table 2). It is worth noting that these novel mutations are mostly private and only two alleles, p.S2251X and p.1912_1912delfs, were observed in two probands. *GPR98* is the second most frequently mutated gene in our USH II patients, with pathogenic mutations occurring in 3 patients. Two homozygous mutations in USH type III gene *CLRN1* were found in 2 USH II patients. Compound heterozygous missense variants in USH type I gene *MYO7A* was identified in USH II patient USHsrf40, who carries two missense variants c.4951G > A: p.D1651N and c. 4360G > A: p.V1454I. Both of these variants are absent in the control database and segregate with disease in the family (Fig. 3). Novel homozygous splicing site mutation c.963 + 1G > A in *DFNB31* was found in a USH II patient from a consanguineous family, which was confirmed by segregation tests.

Patient USHsrf26 was the only patient in our cohort with USH type III, while patient USHsrf3 that does not fit well in any Usher subtype. Patient USHsrf3 was a student in a boarding school for disabled kids. There is no detailed medical record of him and his guardian, a teacher, didn’t know his past medical history. The primary diagnosis of Usher syndrome was reached because he had hearing problems and a retinal phenotype.

No putative mutations in known USH disease genes have been found for either of these two patients.

### Biallelic mutations in 3 patients were found in retinal disease genes previously un-associated with USH

We reasoned that mutations in other known retinal disease genes might account for the clinical phenotype observed in some of the patients in our cohort for several reasons. First, some of our patients may have a different syndromic disease whose phenotype is similar to USH. Second, it other retinal disease genes may cause USH syndrome. Third, the hearing loss and visual defects could co-occur in one individual as a result of mutations in multiple genes, one causing eye disease and a second gene causing hearing defects. Finally, it is possible that only part of the clinical phenotype of a patient has genetic cause.

To test these hypotheses, we checked whether our patients bear mutations in other known retinal disease genes. Interestingly, two patients, USHsrf62 and USHsrf5, were found to carry mutations in *EYS* that have been associated with RP [25]. Patient USHsrf62 is homozygous for a novel frameshift mutation c.910delT:p.W304fs in *EYS* (Additional file 1: Table S5). Patient USHsrf5 carries a novel frameshift mutation, c.8392delG:p.D2798fs and a known missense mutation in *EYS.* Therefore, it is likely that the RP phenotype in these two patients is due to their mutations in *EYS*. As many patients with mutations in *EYS* have been reported and none of them showed hearing loss, it is likely that these patients’ hearing problems are independent of the retinal phenotype. We did observe a splice site mutation and a missense mutation in *LOXHD1* in USHsrf62 in our whole exome sequencing data. Mutations in this gene are associated with non-syndromic hearing loss, making it likely that the patient’s visual and auditory problems have independent genetic origins.

Our molecular diagnosis also suggests that patient USHsrf40’s hearing loss and RP might be explained by mutations in multiple genes. This patient was diagnosed with USH II based on his clinical phenotype. Interestingly, compound heterozygous mutations were identified in both *MYO7A* and *CNGA1* (Fig. 3). Patients with mutations in *MYO7A* exhibited a spectrum of phenotypes ranging from USH I to USH III to atypical USH consisting of non-syndromic hearing loss without retinal a phenotype [9, 26, 27]. In contrast, mutations in *CNGA1* have only been linked to RP so far [28]. Since patient USHsrf40 didn’t have a vestibular problem, a phenotype observed in both USH I and III type patients, it is possible that the two mutations in *MYO7A* in USHsrf40 only lead to deafness while mutations in *CGNA1* are the underlying cause of the RP phenotype. Supporting this idea, mutations in *MYO7A* identified in this patient were indeed relatively weak. In particular, one of the missense variants,*MYO7A*:c.4360 G > A:p.V1454I, was predicted to be neutral by all functional prediction tools used except CADD [29] (Additional file 1: Table S6). Given that the mutation affects a highly conserved amino acid, was absent from all control databases and segregates with the disease, this variant is was likely to be mildly pathogenic.

### No mutations were identified in CIB2, ABHD12 and HARS

Three of the known USH disease genes, *CIB2*, *ABHD12* and *HARS*, were not included in the capture panel. To achieve a comprehensive screening, we performed whole exome sequencing (WES) on patients who were negative for mutations in known USH disease genes after the target capture sequencing. No mutations were identified in these three USH genes that were not included in our panel, indicating that mutations in these three genes are not major causes of USH in Chinese patients.

### *USH2A* mutation severity determines patient phenotype

**Fig. 4:**
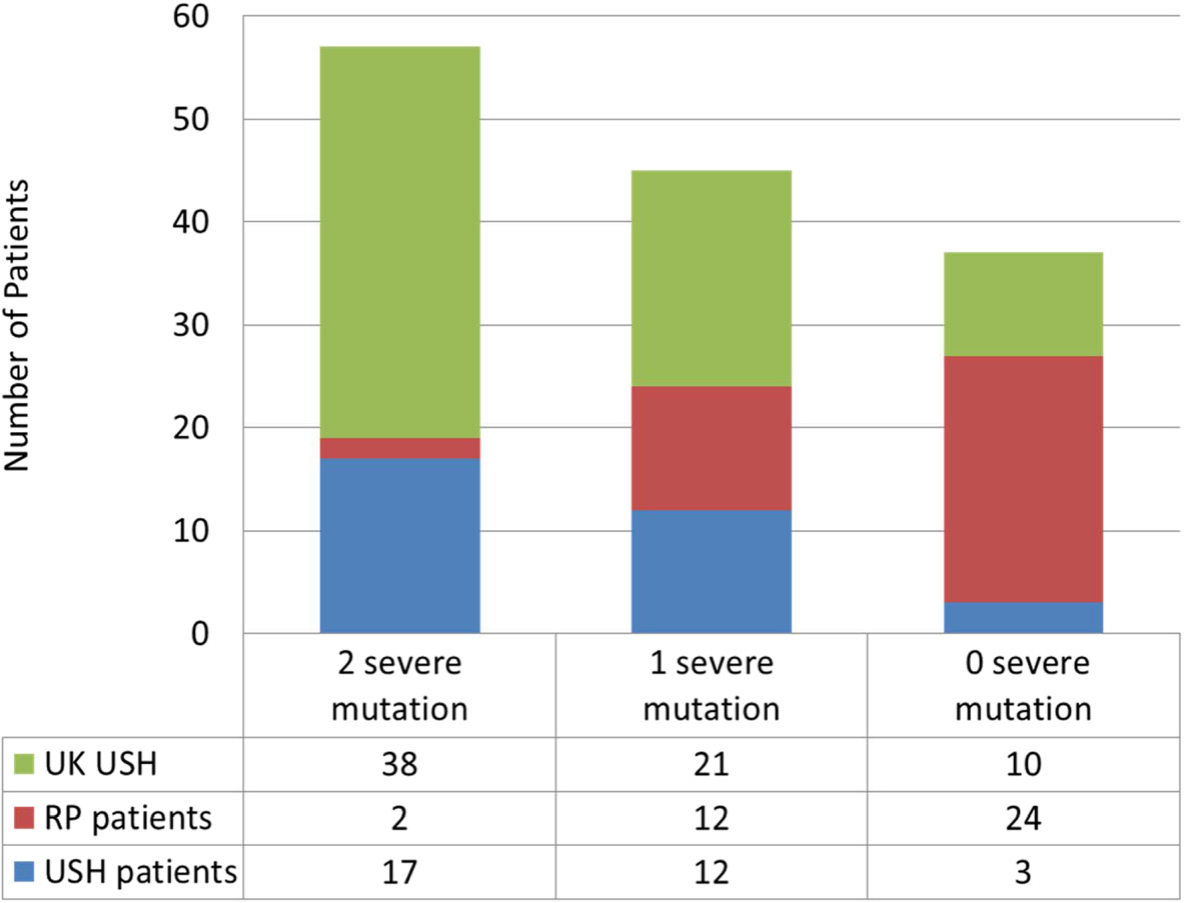
USH patients are highly enriched in patients with two severe alleles. Patients with USH2A mutations were classified based on number of severe alleles (frameshift mutations, splicing site mutations and nonsense mutations). Enrichment of patients with two severe mutations is significant (Fisher exact test, *p*-value < 0.0001) in two independent USH patients cohorts (USH patients in this study [30]) compared to that of RP patients

We identified 40 distinct *USH2A* alleles in this study. Previous studies from multiple groups, including ours, have already shown that mutations in *USH2A* can lead to either USH II or non-syndromic RP [10, 29]. We compared the *USH2A* alleles from 32 USH II patients identified in this paper with a collection of 38 RP patients whose disease was caused by *USH2A* mutations ([10] and our unpublished data). The number of obviously null alleles (including nonsense mutations, splicing mutations, and frameshift mutations) carried by each patient was counted. As shown in Fig. 4, the vast majority of USH II patients carry at least one null allele (29/32). Specifically, 17 patients carry two null alleles and 12 USH II patients carry one null allele. In contrast, among the 38 RP patients, only 2 carry two null alleles and 12 carry one null allele. Therefore, mutations carried by USH II patients tend to be more severe than those found in RP patients (Fisher’s exact test p-value < 0.0001). Indeed, patients with two severe mutations in *USH2A* were predominantly USH II patients (53 % USH II vs 5 % RP) while patients with two missense mutations were largely RP patients (9 % USH II vs 63 % RP). Further supporting our observations, the vast majority of *USH2A* alleles identified from another published USH II patient cohort are null alleles (Fig. 4) [30]. It is likely that severe disruption of *USH2A* causes both hearing and RP phenotypes in most cases, while milder disruptions to *USH2A* only cause RP except in patients with a background or environment predisposed to hearing loss.

## Discussion

In this report, we comprehensively screened 67 unrelated USH families for disease causing mutations. This is the largest Chinese USH cohort molecularly tested to date. All known USH disease genes as well and other known retinal disease genes were screened for mutations using a combination of panel capture and whole exome sequencing, representing the first NGS based comprehensive molecular characterization of a large Usher patient cohort. This approach allowed us to obtain accurate estimates of mutation frequency in known USH disease genes in the Chinese population. Our results reveal a similar but distinct mutation spectrum in Chinese USH patients compared to European patients.

In our cohort, causal mutations were identified in 47 families (49 patients), achieving an overall solving rate of 70 %. This is similar but slightly lower than that of previous studies on patients of European descent, which used sanger sequencing of 9 genes to obtain diagnosis rates of 72 and 85 % [12, 30]. In our patient cohort, *USH2A* and *MYO7A* are the most frequently mutated genes, accounting for 46 and 12 % patients, respectively. This rate is similar to the 55 and 14 %, respectively, observed in a previous study that Sanger sequenced 9 USH genes in 172 ethnically heterogeneous UK patients who are primarily Caucasian [30]. In addition to these two most frequently mutated genes, mutations in *CDH23*, *PCDH15*, *USH1C*, *USH1G*, *GPR98*, *DBNF31*, *CLRN1*, *CIB2*, *ABHD12* and *HARS* have been reported to cause USH syndrome. However, these 10 genes only each account for a small percent of the patients and together account for no more than 20 % [1, 12, 30]. Until now, patients with mutations in these less frequently mutated genes have not been reported for Chinese USH patients. In this cohort, mutations in these genes together account for about 11.4 % patients. Significantly, we identify the first Chinese patient(s) with mutations in*CLRN1*, *DFNB31*, *GPR98* and *PCDH15*.

In contrast to the similarity in frequency of mutations in known USH disease genes between ethnic groups, many alleles identified in the Chinese patient cohort are absent from other ethnic groups. For example, in our study, a total of 40 alleles have been identified in *USH2A* with 67.5 % being novel alleles. This is striking as a large number of mutations (>200) have already been reported for *USH2A* with much lower rate (40 % and 48 %) of novel causal alleles identified in studies screened USH genes in European patients [12, 30]. Since the vast majority of reported alleles come from studies of patients of European descent, the allele spectrum in our Chinese patients differs from that present in current allele databases. This idea is further supported by the observation that, for the 13 known mutations in *USH2A* identified in this study, only 8 alleles were been previously reported in Caucasians, while the other 5 were exclusively reported in Chinese or Japanese patients [16, 18, 31]. Furthermore, strong founder effect has been observed in both ethnic groups. For example, *USH2A*:c.2299delG, which is the most prevalent European mutation and accounts at approximately 30 % of all European *USH2A* [19], was not detected in our patients despite being thoroughly tested. In contrast, the splice site mutation *USH2A:*c.8559-2A > G, which has been previously identified solely in Chinese and Japanese patients, is the most frequent mutation in our patient cohort and was observed in 11 patients.

Our study clearly demonstrates that the molecular basis of USH syndrome is highly heterogeneous in several ways. First, although founder mutations have been identified, the vast majority of the alleles are rare and each only appears in a small fraction of patients. Indeed, 77 % of the alleles identified in our study are novel. The large diversity of mutations in USH genes has also been noted in other Middle Eastern populations [32]. With this in mind, we expect a high rate of novel mutations in the Chinese population. Second, multiple genes have been associated with USH syndrome. To date, 15 USH associated genes have been identified. In our study, mutations in 6 known USH disease genes were found. Third, mutations in the same gene can lead to different clinical phenotypes. For example, CLRN1 mutations have been associated with USH III [23] while in our study, 3 patients with CLRN1 mutations exhibit USH I or USH II. Our study shows CLRN1 causes a broad spectrum of hearing and retinal phenotypes. Finally, the same clinical phenotype can be caused by mutations in multiple genes. For example, one patient in our cohort, USHsrf40, carries mutations in both *MYO7A* (c.4951G > A and c.4360G > A) and *CNGA* (c.265delC and c.479C > T) that result in hearing and vision impairment respectively; therefore this patient does not have canonical USH syndrome. Given this heterogeneity, it is important to combine a patient’s clinical information with their molecular diagnosis in order to provide patients with better prognoses and to help match management and treatment strategies with the patient disease.

Several attempts to establish genotype and phenotype correlation in *USH2A* have been reported [33, 34]. None of these studies identified apparent genotype/phenotype correlations. Similar to previous reports, no apparent genotype/phenotype correlations were observed when we cross referenced the patient’s alleles and their clinical phenotypes. Interestingly, a strong correlation between he genotype and phenotype was observed when we compared alleles obtained from our USH cohort and our own and previously reported RP cohorts. Our study suggests that more severe loss-of-function mutations in *USH2A* lead to syndromic retinopathy. By determining the severity of various USH mutations we could predict the disease that babies or fetuses with a given genotype are likely to develop.

In our patient cohort we observed an enrichment of mono-allelic mutations genetically undiagnosed patients, particularly in *USH2A*. For patients where bi-allelic mutations could not be found, mono-allelic *USH2A* nonsense mutations, frameshift and splicing site mutations occurred in 10 out of 54 USH II patients (20 %) (Additional file 1: Table S4). This is significantly higher than what is observed in controls, which typically have a frequency of less than 1 % (internal unpublished data). This data suggests that a significant portion of *USH2A* mutations might be missed by exome capture sequencing. Given that the entire coding region of the *USH2A* was well covered by our design, it is likely that the missing alleles are either in non-coding regions or are structural rearrangements, such as deletions or inversions that affect *USH2A* protein production. Indeed, a recent study of the*USH2A* locus reveals 35 % of *USH2A* monoallelic cases can be solved by screening for duplications, deletions and deep intronic mutations [35]. Thus, much of the missing heritability in USH could be achieved as follows. First, we could discover mutations not previously annotated by improving functional prediction software. An example of this is a recent study of *ABCA4* that demonstrated the effect of synonymous mutations and splice site modification mutations as a major cause of Stargardt’s disease [36]. Second, we can use a compensatory method, such as comparative genomic hybridization, to detect duplications and deletions. Third, sequencing gene promoters and other regulatory regions will allow for the identification of pathogenic regulatory mutations. The lack of reliable prediction tools and high throughput experimental assays are the main bottlenecks in identifying these types of mutations.

In summary, we report the first NGS-based comprehensive molecular survey of a large Chinese USH patient cohort. Our results suggest that up to 90 % of USH patients are due to mutations in known USH disease genes when including patients with monoallelic mutations in *USH2A*. By combining molecular diagnosis and patient clinical information, a more accurate diagnosis, prognoses and personalized treatment of individual USH patients can be achieved.

## Conclusions

Our study provides the first comprehensive characterization of a large collection of Chinese USH patients. Up to 90 % of USH patients are due to mutations in known USH disease genes. By combing NGS-based molecular diagnosis and patient clinical information, a more accurate diagnosis, prognosis and personalized treatment of USH patients can be achieved.

## Additional files

Additional file 1: Table S1. Genes in the panel design. **Table S2.** Summary of coverage for coding exons and flanking regions (+ 2bp). **Table S3.** Clinical information of patients. **Table S4.** High confidence monoallelic mutations in *USH2A* genes*. **Table S5.** Rare biallelic variants in other eye disease genes*. **Table S6.** In-silico function prediction of nonsynonymous variants (DOCX 80 kb) Additional file 2: Figure S1. Both Novel missense alleles identified in MYO7A genes are conserved between human, zebrafish and *Drosophila melanogaster*. (PPTX 676 kb)

## Abbreviations

USH: USH syndrome
ERG: Electroretinograms
OCT: Optical coherence tomography
NGS: Next generation sequencing
RP: Retinitis pigmentosa
BCVA: Best-corrected visual acuity
