# Supplementary material for: Comprehensive molecular diagnosis of 67 Chinese Usher syndrome probands: high rate of ethnicity specific mutations in Chinese USH patients
Source: Orphanet J Rare Dis. 2015 Sep 4;10:110. doi: 10.1186/s13023-015-0329-3 (PMC4559966; doi:10.1186/s13023-015-0329-3)
Supplement: Additional file 1: Table S1. — Genes in the panel design. Table S2. Summary of coverage for coding exons and flanking regions (+ 2bp). Table S3. Clinical information of patients. Table S4. High confidence monoallelic mutations in USH2A genes*. Table S5. Rare biallelic variants in other eye disease genes*. Table S6. In-silico function prediction of nonsynonymous variants (DOCX 80 kb) [file 13023_2015_329_MOESM1_ESM.docx]

Table S1. Genes in the panel design

|  |  |  |  |  |  |  |  |  |  |  |  |  |  |  |  |
| --- | --- | --- | --- | --- | --- | --- | --- | --- | --- | --- | --- | --- | --- | --- | --- |
| 10 genes known to be causative or relevant to Usher syndrome | | | | | |  |  |  |  |  |  |  |  |  |  |
| CDH23 | CLRN1 | DFNB31 | GPR98 | MYO7A | PCDH15 | PDZD7 | USH1C | USH1G | USH2A |  |  |  |  |  |  |
|  |  |  |  |  |  |  |  |  |  |  |  |  |  |  |  |
| 64 genes known to be causative for retinitis pigmentosa | | | | |  |  |  |  |  |  |  |  |  |  |  |
| CDH23 | CLRN1 | DFNB31 | MYO7A | PCDH15 | USH1C | USH2A | ABCA4 | BEST1 | C2ORF71 | C8ORF37 | CA4 | CC2D2A | CERKL | CNGA1 |  |
| CNGB1 | CRB1 | CRX | DHDDS | EYS | FAM161A | FLVCR1 | FSCN2 | GNPTG | GUCA1B | IDH3B | IMPDH1 | IMPG2 | KLHL7 | LRAT |  |
| MAK | MERTK | NR2E3 | NRL | PANK2 | PDE6A | PDE6B | PDE6G | PGK1 | PRCD | PROM1 | PRPF3 | PRPF31 | PRPF6 | PRPF8 |  |
| PRPH2 | RBP3 | RDH12 | RGR | RHO | RLBP1 | ROM1 | Rp1 | Rp2 | Rp8 | RPE65 | RPGR | SAG | SEMA4A | SNRNP200 |  |
| TOPORS | TTC8 | TTPA | TULP1 |  |  |  |  |  |  |  |  |  |  |  |  |
|  |  |  |  |  |  |  |  |  |  |  |  |  |  |  |  |
| 19 genes know to be causative for Leber congenital amaurosis | | | | | |  |  |  |  |  |  |  |  |  |  |
| AIPL1 | CABP4 | CEP290 | CRB1 | CRX | GUCY2D | IMPDH1 | IQCB1 | KCNJ13 | LCA5 | LRAT | NMNAT1 | OTX2 | RD3 | RDH12 |  |
| RPE65 | RPGRIP1 | SPATA7 | TULP1 |  |  |  |  |  |  |  |  |  |  |  |  |
|  |  |  |  |  |  |  |  |  |  |  |  |  |  |  |  |
| 11 genes know to be causative for congenital stationary night blindness | | | | | | |  |  |  |  |  |  |  |  |  |
| CABP4 | CACNA1F | GNAT1 | GPR179 | GRK1 | GRM6 | NYX | PDE6B | RHO | SLC24A1 | TRPM1 |  |  |  |  |  |
|  |  |  |  |  |  |  |  |  |  |  |  |  |  |  |  |
| 41 other genes cause syndromic eye disease | | | |  |  |  |  |  |  |  |  |  |  |  |  |
| AHI1 | ALMS1 | ARL6 | BBS1 | BBS10 | BBS12 | BBS2 | BBS4 | BBS5 | BBS7 | BBS9 | CISD2 | CNNM4 | COL11A1 | COL2A1 |  |
| COL9A1 | ERCC6 | INPP5E | INVS | JAG1 | LRP5 | MFRP | MKKS | NPHP1 | NPHP3 | NPHP4 | OFD1 | PAX2 | PDZD7 | Rp9 |  |
| RPGRIP1L | TIMM8A | TMEM126A | TMEM216 | TMEM237 | TREX1 | TRIM32 | VPS13B | WDPCP | WFS1 | WDPCP |  |  |  |  |  |
|  |  |  |  |  |  |  |  |  |  |  |  |  |  |  |  |
|  |  |  |  |  |  |  |  |  |  |  |  |  |  |  |  |
| 69 other types of eye disease gene | | |  |  |  |  |  |  |  |  |  |  |  |  |  |
| ABCC6 | ADAM9 | ARMS2 | ATXN7 | C1QTNF5 | C2 | C3 | CACNA2D4 | CDH3 | CDHR1 | CEP164 | CFB | CFH | CHM | CLN3 | CNGA3 |
| CNGB3 | CYP4V2 | DMD | EFEMP1 | ELOVL4 | FBLN5 | FZD4 | GNAT2 | GUCA1A | HMCN1 | HTRA1 | KCNV2 | KIF11 | MFN2 | MKS1 | MPV17 |
| MTTP | NDP | OAT | OPA1 | OPA3 | OPN1LW | OPN1MW | OPN1MW2 | OPN1SW | PDE6C | PEX1 | PEX2 | PEX7 | PHYH | PITPNM3 | PLA2G5 |
| PRD | PXMP3 | RAX2 | RB1 | RBP4 | RCD1 | RDH5 | RGS9 | RGS9BP | RIMS1 | RP1L1 | RRM2B | RS1 | SDCCAG8 | SLC6A5 |  |
| TEAD1 | TIMP3 | TK2 | TLR3 | TLR4 | TSPAN12 | UNC119 | VCAN | WDR19 |  |  |  |  |  |  |  |

## Table S2. Summary of coverage for coding exons and flanking regions (+ 2bp).

| genes | average rate  of 10X coverage | minimum rate  of 10X coverage | average rate  of 20X coverage | minimum rate  of 20X coverage | Mean  coverage |
| --- | --- | --- | --- | --- | --- |
| all gene | 96.8 | 93.9 | 94.6 | 86.7 | 109 |
| *CDH23* | 99.5 | 95.6 | 97.6 | 82.3 | 87.6 |
| *CLRN1* | 96.8 | 93.7 | 95.3 | 88.5 | 93.2 |
| *DFNB31* | 90.8 | 83.1 | 86 | 63.1 | 69.7 |
| *GPR98* | 99.8 | 99.8 | 99.8 | 98.9 | 96.9 |
| *MYO7A* | 95.2 | 87.3 | 90.6 | 67.4 | 78.2 |
| *PCDH15* | 99.4 | 98.2 | 98.4 | 95.1 | 95.4 |
| *PDZD7* | 89.1 | 58.1 | 77.8 | 32 | 55.1 |
| *USH1C* | 96.9 | 83.1 | 91.9 | 56.7 | 73 |
| *USH1G* | 93.2 | 74.9 | 79.4 | 43.4 | 49.1 |
| *USH2A* | 100 | 100 | 99.9 | 99.2 | 126.2 |

Table S3. Clinical information of patients

| Patient ID | Family ID | sex | age | USH type | visual acuity | time of vision loss onset | time of hearing loss onset | lens and fundus variation | OCT | VF | ERG |
| --- | --- | --- | --- | --- | --- | --- | --- | --- | --- | --- | --- |
| USHsrf11 | 1 | M | 46 | II | 0.03/0.03 | school age | school age | accepted cataract surgery, pigmentation variation in the midperiphery of retina |  |  |  |
| USHsrf63 | 2 | M | 53 | II | 0.05/0.05 | 8 | school age | postcapsular opacity of lens, pigmentation variation as bone spicule in the midperiphery of retina | atrophy of both neural epithelium layer |  | unrecordable |
| USHsrf23 | 3 | M | 33 | II | 0.06/0.06 | 20 | school age | pigmentation variation as grey and bone spicule in the midperiphery |  |  |  |
| USHsrf59 | 4 | M | 57 | II | 0.06/0.06 | teenage | school age | postcapsular opacity of lens, attenuation of retinal vascular, pigmentation variation as bone spicule in the midperiphery of retina |  |  |  |
| USHsrf66 | 4 | M | 31 | II | 0.8/0.5 | 12 | 5 | attenuation of retinal vascular, pigmentation variation as bone spicule in the midperiphery of retina |  |  |  |
| USHsrf68 | 4 | F | 61 | II | HM/LP | childhood | childhood | mild opacity of right eye,left aphakia eye and opacity of postcapsular, pigmentation variation in the midperiphery of retina |  |  |  |
| USHsrf21 | 5 | M | 43 | II | 0.1/0.08 | childhood | 7 | postcapsular opacity of lens, pigmentation variation as bone spicule,salt and pepper in midperiphery of retina,both optic disk are pale | neural epithelium layer is atrophy, lack of IS/OS |  |  |
| USHsrf56 | 6 | F | 38 | I | 0.1/0.1 | 8 | 7 | pigmentation variation in the midperiphery | the whole retina became thinner |  |  |
| USHsrf45 | 7 | F | 50 | II | 0.2/0.2 | 16 | school age | postcapsular opacity of lens, pigmentation variation in the midperiphery of retina | neural epithelium layer became thinner, lack of IS/OS, atrophy of RPE | tunnel vision | unrecordable |
| USHsrf46 | 8 | F | 36 | II | 0.2/0.6 | 15 | childhood | cortex opacity of lens,pigmentation variation as grey in the midperiphery of retina |  |  |  |
| USHsrf61 | 9 | M | 37 | I | 0.3/0.05 | 3 | childhood | postcapsular opacity of lens, pigmentation variation as bone spicule in the whole retina | Lack of IS/OS and atrophy of RPE |  |  |
| USHsrf1 | 10 | M | 26 | II | 0.3/0.3 | 15 | 10 | grey granules and bone spicule in peripheral retina |  | central field |  |
| USHsrf35 | 11 | F | 22 | II | 0.3/0.3 | 10 | 6 | attenuation of retinal vascular, pigmentation variation as bone spicule in the midperiphery of retina |  | central bright |  |
| USHsrf9 | 12 | M | 51 | II | 0.3/0.5 | 10 | 10 | postcapsular opacity of lens, pigmentation variation in the whole retina | macular fovea became thinner, atrophy of RPE |  |  |
| USHsrf37 | 13 | M | 31 | II | 0.4/0.15 | 15 | 27 | pigmentation variation as grey and bone spicule in the whole retina | neural epithelium layer became thinner, lack of IS/OS |  |  |
| USHsrf36 | 14 | M |  | II | 0.4/0.2 | 30 | 36 | postcapsular opacity of lens, pigmentation variation as bone spicule in the midperiphery,reflection in the macula as gold foil | the whole retina became thinner,shape of macular fovea disappeared,lack of IS/OS, atrophy of RPE |  |  |
| USHsrf7 | 15 | M | 25 | II | 0.4/0.4 | 15 | 10 | pigmentation variation as bone spicule in midperiphery of retina | RPE atrophy |  | unrecordable |
| USHsrf14 | 16 | F | 22 | I | 0.4/0.4 | school age | school age | pigmentation variation as grey in the midperiphery of retina, enhancement of relection of macular | lack of IS/OS ,apophysis of macular fovea | tunnel vision | rod response unrecordable, cone response unrecordable |
| USHsrf17 | 17 | M | 11 | I | 0.4/0.4 | 9 | childhood | pigmentation variation as grey in the midperiphery of retina, enhancement of relection of macular |  |  | rod response unrecordable, cone response unrecordable |
| USHsrf18 | 18 | F | 33 | II | 0.4/0.4 | school age | childhood | postcapsular opacity of lens,attenuation of retinal vascular,pigmentation variation as grey in the midperiphery of retina |  |  |  |
| USHsrf24 | 19 | F | 30 | II | 0.5/0.3 | 18 | 7 | attenuation of retinal vascular,pigmentation variation as grey in the midperiphery of retina | neural epithelium layer became thinner |  |  |
| USHsrf25 | 19 | M | 50 | II | HM/0.1 | 20 | 40 | opacity of lens and pigmentation variation as bone spicule in the midperiphery of retina |  |  |  |
| USHsrf2 | 20 | M | 40 | II | 0.5/0.6 | 15 | 6 | postcapsular opacity of lens, pigmentation variation as salt and pepper in midperiphery of retina | neural epithelium layer became thinner and lack IS/OS except macular fovea | tunnel vision | unrecordable |
| USHsrf20 | 21 | M | 37 | II | 0.5/0.7 | 15 | 27 | pigmentation variation as grey in the midperiphery and plaque in macular | macular fovea became thinner and RPE layer became thinner irregularly, IS/OS layer are unclear | tunnel vision |  |
| USHsrf38 | 22 | M | 33 | II | 0.6/0.7 | teenage | 29 | pigmentation variation as salt and pepper in midperiphery of retina | neural epithelium layer became thinner ,epiretinal membrane and lack IS/OS | tunnel vision | both cone and rod response reduced |
| USHsrf32 | 23 | M |  | II | 0.6/0.8 | 15 | 20 | postcapsular opacity of lens, pigmentation variation as bone spicule in the midperiphery of retina | neural epithelium layer and the macular became thinner | pangzhongxintouliangzuoshiyequesun | rod response:right, reduced;left, unrecordable. Cone response reduced |
| USHsrf47 | 24 | M |  | II | 0.6/LP | 15 | 5 | postcapsular opacity of lens, pigmentation variation as bone spicule in the midperiphery of retina, glaucoma |  | central bright |  |
| USHsrf70 | 25 | F | 29 | II | 0.7/0.4 | 23 | 3 | pigmentation variation as grey and bone spicule in the midperiphery,highly reflection of macular | macular edema |  |  |
| USHsrf8 | 26 | M | 17 | I | 0.7/0.5 | school age | school age | pigmentation variation in midperiphery of retina | macular edema, lack of IS/OS except macular fovea | tunnel vision |  |
| USHsrf22 | 27 | M | 12 | I | 0.8/0.8 | school age | childhood | pigmentation variation in midperiphery of retina | retinoschisis |  | rod response reduced |
| USHsrf10 | 28 | M | 22 | II | 0.9/0.7 | school age | 1 | postcapsular opacity of lens, pigmentation variation as grey in the midperiphery and reflection as gold foil in the macular area | neural epithelium layer and RPE layer are atrophy, lack of IS/OS |  | rod response unrecordable, cone response reduce |
| USHsrf60 | 29 | M | 5 | II | 1.0/0.9 | 20 | school age | pigmentation variation as salt and pepper in midperiphery of retina |  |  |  |
| USHsrf33 | 30 | F | 21 | II | 1.0/1.0 | 10 | 3 | pigmentation variation in the midperiphery of retina |  |  |  |
| USHsrf69 | 31 | M | 42 | II | 1.0/1.2 | 35 | 32 | pigmentation variation as grey and bone spicule in the midperiphery of retina |  |  | rod response unrecordable, cone response reduce |
| USHsrf62 | 32 | M | 29 | II | 0.7/0.6 | 15 | 25 | postcapsular opacity of lens and pigmentation variation in the midperiphery of retina |  |  |  |
| USHsrf13 | 33 | F | 37 | II | 0.6/0.4 | 20 | teenage | pigmentation variation as salt and pepper in midperiphery of retina |  |  |  |
| USHsrf57 | 34 | M | 24 | II | 0.8/1.0 | 22 | 21 | pigmentation variation as grey in the midperiphery |  |  |  |
| USHsrf58 | 35 | M | 27 | II | 0.3/0.4 | teenage | 3 | postcapsular opacity of lens and pigmentation variation as bone spicule in the midperiphery of retina |  |  |  |
| USHsrf26 | 36 | M | 59 | III | o.2/0.2 | 40 | 50+ | nuclear and postcapsular opacity of lens and pigmentation variation as bone spicule in the midperiphery of retina |  |  |  |
| USHsrf65 | 37 | M | 55 | II | 0.2/0.2 | teenage | 7 | postcapsular opacity of lens and pigmentation variation as bone spicule in the midperiphery of retina | neural epithelium layer became thinner and lack IS/OS | tunnel vision | rod response unrecordable, cone response unrecordable |
| USHsrf12 | 38 | F | 53 | II | 0.01/0.01 | teenage | teenage | postcapsular opacity of lens and pigmentation variation as bone spicule in the midperiphery of retina |  |  |  |
| USHsrf15 | 39 | F | 35 | II | 0.6/0.2 | 30+ | 15 | intraocular lens is normal, macular become red, pigmentation variation as bone spicule in the midperiphery of retina, thinner of retinal artery | neural epithelium layer became thinner and lack IS/OS ,atrophy of RPE | central bright | rod response unrecordable, cone response unrecordable |
| USHsrf4 | 40 | F | 35 | II | 0.6/0.6 | teenage | teenage | postcapsular opacity of lens and pigmentation variation in the midperiphery of retina | neural epithelium layer became thinner and lack IS/OS ,atrophy of RPE | central bright | rod response unrecordable, cone response unrecordable |
| USHsrf28 | 41 | M | 24 | I | 1.0/1.0 |  | school age | pigmentation variation in the midperiphery of retina | lack IS/OS and thinner neural epithelium layer |  |  |
| USHsrf30 | 42 | M | 60 | II | 0.25/0.4 | 40 | teenage | postcapsular opacity of lens of left eye and pigmentation variation as bone spicule in the midperiphery of retina of both eye,atrophy of RPE | lack IS/OS and atrophy of RPE | tunnel vision | rod response unrecordable, cone response unrecordable |
| USHsrf3 | 43 | M | 22 | NA |  |  |  |  |  |  |  |
| USHsrf5 | 44 | F | 61 | II | NLP/LP | 10 | 40 | anterior capsular and postcapsular opacity of lens and pigmentation variation in the midperiphery of retina |  |  |  |
| USHsrf6 | 45 | M | 24 | I | 0.8/0.3 | 19 |  | pigmentation variation as bone spicule in the midperiphery of retina | right eye，epiretinal membrane of macular and discontinuous of IS/OS, left eye,neural epithelium layer became thinner and lack IS/OS | tunnel vision | rod response unrecordable, cone response unrecordable |
| USHsrf16 | 46 | F | 35 | II | 1.0/1.0 | 33 | school age | enhancement of reflection in posterior retina and pigmentation variation near the vascular | neural epithelium layer became thinner and atrophy of RPE | central bright | rod response unrecordable, cone response unrecordable |
| USHsrf19 | 47 | F | 33 | II | 0.5/0.4 | 17 | school age | pigmentation variation as bone spicule and grey in the midperiphery of retina, thinner of retinal artery | lack IS/OS and atrophy of RPE | infratemporal bright | rod response unrecordable, cone response unrecordable |
| USHsrf27 | 48 | M | 10 | I | 0.2/0.2 | childhood | childhood | pigmentation variation in the midperiphery of retina | lack IS/OS |  |  |
| USHsrf29 | 49 | M | 40 | II | 0.3/0.4 | teenage | teenage | pigmentation variation as bone spicule in the midperiphery of retina | lack IS/OS except macular fovea | tunnel vision |  |
| USHsrf31 | 50 | F | 36 | II | 0.8/1.0 | not yet | 25 |  |  |  |  |
| USHsrf34 | 51 | F | 36 | II | 0.6/0.6 | 16 | 30 | pigmentation variation as bone spicule in the midperiphery of retina and thinner of retinal vessels | thinner neural epithelium layer, lack of IS/OS except macular fovea and atrophy of RPE | tunnel vision | rod response unrecordable, cone response unrecordable |
| USHsrf39 | 52 | M | 7 | I | 0.6/0.5 | 3 | school age | pigmentation variation as salt and pepper in midperiphery of retina |  |  |  |
| USHsrf40 | 53 | F | 56 | II | NLP/0.5 | 15 | 25 | opacity of lens, cup/disc ratio: right,0.9; left,0.4. pigmentation variation in the midperiphery of retina |  |  |  |
| USHsrf41 | 54 | M | 8 | I | 0.4/0.5 | school age | 2 |  |  |  |  |
| USHsrf42 | 55 | F | 42 | II | 0.25/0.25 | 10 | 7 | pigmentation variation as bone spicule in the midperiphery of retina |  |  |  |
| USHsrf43 | 56 | M | 44 | II |  | teenage | teenage |  |  |  |  |
| USHsrf44 | 57 | F | 43 | I | 0.25/0.25 | childhood | childhood | pigmentation variation as bone spicule in the midperiphery of retina and thinner of retinal vessels |  |  |  |
| USHsrf48 | 58 | f | 50 | II | 0.5/0.01 | 25 | childhood | pigmentation variation as bone spicule in the midperiphery of retina and thinner of retinal vessels | thinner neural epithelium layer, lack of IS/OS except macular fovea and atrophy of RPE | tunnel vision |  |
| USHsrf49 | 59 | M | 57 | II | 1.0/1.0? | 40 | 40 | pigmentation variation in the midperiphery of retina | discontinuous of IS/OS layer and atrophy of RPE |  | rod response reduced, cone response reduced |
| USHsrf50 | 60 | F | 27 | II | 0.8/0.8 | 14 | childhood | grey pigmentation variation as in the midperiphery of retina | little cavity under macular fovea, lack of IS/OS except macular fovea and atrophy of RPE |  | rod response unrecordable, cone response unrecordable |
| USHsrf51 | 61 | f | 62 | II | LP/LP | 40 | teenage | pigmentation variation in the whole retina and thinner of retinal vessels | thinner neural epithelium layer, lack of IS/OS and atrophy of RPE |  |  |
| USHsrf52 | 62 | M | 46 | II | 0.5/0.5 | 20 | 17 | pigmentation variation as bone spicule in the midperiphery of retina and thinner of retinal vessels | thinner neural epithelium layer, lack of IS/OS | tunnel vision | rod response unrecordable, cone response unrecordable |
| USHsrf53 | 63 | F | 26 | I | CF/CF | childhood | childhood | pigmentation variation as bone spicule in the whole retina | thinner neural epithelium layer, lack of IS/OS and atrophy of RPE |  |  |
| USHsrf54 | 64 | M | 47 | II | 0.1/0.25 | 15 | 40 | thinner of retinal vessels, leopard fundus | thinner neural epithelium layer, lack of IS/OS and atrophy of RPE |  |  |
| USHsrf55 | 65 | f | 27 | II | 1.0/0.6 | 15 | 15 | pigmentation variation as bone spicule in the midperiphery of retina and thinner of retinal vessels, leopard fundus | shape of macular fovea disappeared, discontinuous of IS/OS layer | tunnel vision |  |
| USHsrf64 | 66 | F | 47 | II | 0.1/CF | school age | school age | pigmentation variation as bone spicule and grey in the whole retina | thinner neural epithelium layer, lack of IS/OS and atrophy of RPE |  |  |
| USHsrf67 | 67 | F | 31 | II | 0.5/0.5 | childhood | childhood | pigmentation variation in the midperiphery of retina | thinner neural epithelium layer of macular and lack of IS/OS |  |  |

Table S4. High confidence monoallelic mutations in *USH2A* genes*

| patient | ush type | gene | type | NMID | exon | cDNA | amino acid | genotype | patient origin | reference |
| --- | --- | --- | --- | --- | --- | --- | --- | --- | --- | --- |
| USHsrf4 | II | USH2A | Splicing | NM_206933 | 59 | c.11389+1G>C | c.11389+1G>C | Heterozygous | Chinese | Novel |
| USHsrf6 | I | USH2A | frameshift | NM_206933 | 40 | c.7521_7522insT | p.M2507fs | Heterozygous | Chinese | Novel |
| USHsrf13 | II | USH2A | Splicing | NM_206933 | 44 | c.8559-2A>G | c.8559-2A>G | Heterozygous | Japanese | [[1](#_ENREF_1)] |
| USHsrf16 | II | USH2A | Splicing | NM_206933 | 44 | c.8559-2A>G | c.8559-2A>G | Heterozygous | Japanese | [[1](#_ENREF_1)] |
| USHsrf29 | II | USH2A | stopgain | NM_206933 | 49 | c.C9723A | p.Y3241X | Heterozygous | Chinese | Novel |
| USHsrf34 | II | USH2A | Splicing | NM_206933 | 44 | c.8559-2A>G | c.8559-2A>G | Heterozygous | Japanese | [[1](#_ENREF_1)] |
| USHsrf3 | NA | USH2A | nonsynonymous | NM_206933 | 42 | c.G8232C | p.W2744C | Heterozygous | Chinese | [[2](#_ENREF_2)] |
| USHsrf58 | II | USH2A | Splicing | NM_206933 | 52 | c.10182+1G>A | c.10182+1G>A | Heterozygous | Chinese | Novel |
| USHsrf68 | II | USH2A | Splicing | NM_206933 | 44 | c.8559-2A>G | c.8559-2A>G | Heterozygous | Japanese | [[1](#_ENREF_1)] |
| USHsrf47 | II | USH2A | stopgain | NM_206933 | 49 | c.C9723A | p.Y3241X | Heterozygous | Chinese | Novel |

Table S5. Rare biallelic variants in other eye disease genes*

| sample | USH type | gene | type | NMID | exon | cDNA | amino acid | genotype | patient origin | reference |
| --- | --- | --- | --- | --- | --- | --- | --- | --- | --- | --- |
| USHsrf5 | II | EYS | frameshift | NM_001142800 | 43 | c.8392delG | p.D2798fs | heterozygous | Chinese | Novel |
| USHsrf5 | II | EYS | nonsynonymous | NM_001142800 | 32 | c.G6557A | p.G2186E | heterozygous | south Korean | [[3](#_ENREF_3)] |
| USHsrf40 | II | CNGA1 | frameshift | NM_000087 | 6 | c.265delC | p.L89fs | heterozygous | Chinese | Novel |
| USHsrf40 | II | CNGA1 | nonsynonymous | NM_000087 | 9 | c.C479T | p.P160L | heterozygous | Chinese | Novel |
| USHsrf62 | II | EYS | frameshift | NM_001142800 | 6 | c.910delT | p.W304fs | homozygous | Chinese | Novel |

| *Unless stated otherwise, alleles are not found in any of the database we used for control common variants |
| --- |
| ^^: 1/2184 in 1000 genome |
| $: 0.000227 in ESP6500 |
| #: rs111033280;CLN;PM;LSD |
| ^: 1/2184 in 1000 genome |
| %: 1/2184 in 1000 genome |

Table S6. In-silico function prediction of nonsynonymous variants

| gene | NMID | amino acid | SIFT_score | SIFT | Polyphen2_HDIV_score | Polyphen2_HDIV_pred | Polyphen2_HVAR_score | Polyphen2_HVAR_pred | LRT_score | LRT_pred | MutationTaster_score | MutationTaster_pred | MutationAssessor_score | MutationAssessor_pred |
| --- | --- | --- | --- | --- | --- | --- | --- | --- | --- | --- | --- | --- | --- | --- |
| CDH23 | NM_022124 | p.V1908I | 0.02 | D | 1 | D | 0.299 | B | 0 | D | 0.917565 | D | 3.205 | medium |
| CDH23 | NM_022124 | p.R2956C | 0.34 | T | 0 | B | 1 | D | 0.000139 | D | 0.996207 | D | 2.705 | medium |
| CLRN1 | NM_001195794 | p.G64A | 0 | D | 1 | D | 0.039 | B | 0 | D | 0.999965 | D | 2.845 | medium |
| CLRN1 | NM_052995 | p.Q7K | na | na | na | na | 0.008 | B | 0 | D | 1 | D | 2.71 | medium |
| GPR98 | NM_032119 | p.R2377Q | 0 | D | 1 | D | 0.066 | B | 0 | D | 0.999819 | D | 2.215 | medium |
| GPR98 | NM_032119 | p.G310E | 0.03 | D | 0.996 | D | 0.892 | P | 0.000048 | D | 0.826945 | D | 2.955 | medium |
| GPR98 | NM_032119 | p.E2504G | 0.05 | D | 0.986 | D | 0.997 | D | 0.000233 | N | 0.984045 | D | 2.075 | medium |
| GPR98 | NM_032119 | p.S4350P | 0.23 | T | 0.794 | P | 0.478 | P | 0.000948 | D | 0.282045 | N | 2.525 | medium |
| MYO7A | NM_001127180 | p.Q188E | 0 | D | 1 | D | 0.999 | D | 0.000001 | D | 0.996927 | D | 2.54 | medium |
| MYO7A | NM_001127180 | p.R1168Q | 0 | D | 1 | D | 0.062 | B | 0.000002 | D | 0.999965 | D | 3.92 | high |
| MYO7A | NM_001127180 | p.R395C | 0 | D | 1 | D | 0.786 | P | . | . | 0.997225 | D | 4.48 | high |
| MYO7A | NM_001127180 | p.R405Q | 0.03 | D | 1 | D | 0.018 | B | 0.000076 | D | 0.980587 | D | 2.36 | medium |
| MYO7A | NM_001127180 | p.V1454I | 0.13 | T | 0.054 | B | 0.371 | B | 0.0261 | N | 0.004227 | N | -0.09 | neutral |
| MYO7A | NM_001127180 | p.R2023Q | 0.17 | T | 0.928 | P | . | . | 0.000686 | D | 0.999869 | D | 1.975 | medium |
| MYO7A | NM_001127180 | p.D1651N | 0.27 | T | 0.088 | B | 1 | D | 0 | D | 0.987992 | D | 3.045 | medium |
| MYO7A | NM_001127180 | p.M946R | 0 | D | 0.835 | P | 0.548 | P | 0 | D | 0.99996 | D | 1.935 | medium |
| MYO7A | NM_001127180 | p.L1799P | 0 | D | 1 | D | 0.986 | D | 0.000001 | D | 0.99947 | D | 3.396 | median |
| PCDH15 | NM_001142773 | p.R940C | 0 | D | 0.992 | D | 0.001 | B | 0.001117 | U | 0.289388 | N | 2.015 | medium |
| PCDH15 | NM_001142773 | p.R940C | 0.03 | D | 1 | D | 0.969 | D | . | . | 0.998302 | D | 3.045 | medium |
| PCDH15 | NM_001142773 | p.V1230A | 0.3 | T | 1 | D | 0.003 | B | 0.00087 | D | 0.993642 | D | 2.2 | medium |
| USH1C | NM_153676 | p.S851A | 0 | D | 0.977 | D | 0.031 | B | . | . | 0.955183 | D | 3.625 | high |
| USH1G | NM_173477 | p.L244R | 0.04 | D | 0.996 | D | 0.731 | P | 0.000002 | D | 0.999302 | D | 1.61 | low |
| USH2A | NM_206933 | p.C934W | 0 | D | 1 | D | 0.132 | B | 0.000149 | D | 0.746814 | D | 4.645 | high |
| USH2A | NM_206933 | p.C934W | 0 | T | 1 | D | 1 | D | 0.000149 | D | 1 | D | 4.48 | high |
| USH2A | NM_206933 | p.G1861S | 0 | D | 1 | D | 0.989 | D | 0.275742 | N | 0.620147 | D | 0.49 | neutral |
| USH2A | NM_206933 | p.G268R | 0 | D | 1 | D | 0.92 | D | 0.000254 | D | 0.97407 | D | 1.4 | low |
| USH2A | NM_206933 | p.P3272L | 0 | D | 1 | D | 0.996 | D | 0 | D | 0.99658 | D | 2.57 | medium |
| USH2A | NM_206933 | p.W2744C | 0 | D | 1 | D | 0.999 | D | 0 | D | 0.713718 | D | . | . |
| USH2A | NM_206933 | p.W2744C | 0 | D | 1 | D | 0.998 | D | 0.000686 | D | 0.999783 | D | 3.245 | medium |
| USH2A | NM_206933 | p.R1870W | 0.01 | D | 0.999 | D | 0.052 | B | 0 | D | 0.999943 | D | 2.215 | medium |
| USH2A | NM_206933 | p.R334W | 0.01 | D | 1 | D | 0.559 | P | 0.000001 | D | 0.965623 | D | 0.345 | neutral |
| USH2A | NM_206933 | p.S69I | 0.01 | T | 0.963 | D | 0.839 | P | 0.023257 | U | 0.965523 | D | 2.075 | medium |
| USH2A | NM_206933 | p.C972G | 0.02 | D | 1 | D | 0.013 | B | 0 | D | 0.289388 | N | 1.905 | medium |
| USH2A | NM_206933 | p.S2498C | 0.05 | D | 0.921 | P | . | . | 0.000542 | D | 0.999158 | D | 1.79 | low |
| USH2A | NM_206933 | p.V350I | 0.46 | T | 0.001 | B | 1 | D | 0.088125 | N | 0.978908 | D | 2.47 | medium |
| USH2A | NM_206933 | p.G1734R | 0.5 | T | 0.11 | B | 0.024 | B | 0.010069 | N | 0.962138 | D | 1.375 | low |
| USH2A | NM_206933 | p.G3320C | na | na | 1 | D | 0.054 | B | 0.000002 | D | 0.364692 | N | 1.1 | low |

References:

1. Nakanishi, H., et al., *Identification of 11 novel mutations in USH2A among Japanese patients with Usher syndrome type 2.* Clin Genet, 2009. **76**(4): p. 383-91.

2. Xu, W., et al., *Seven novel mutations in the long isoform of the USH2A gene in Chinese families with nonsyndromic retinitis pigmentosa and Usher syndrome Type II.* Mol Vis, 2011. **17**: p. 1537-52.

3. Littink, K.W., et al., *Mutations in the EYS gene account for approximately 5% of autosomal recessive retinitis pigmentosa and cause a fairly homogeneous phenotype.* Ophthalmology, 2010. **117**(10): p. 2026-33, 2033 e1-7.
