# Supplementary material for: Comprehensive molecular diagnosis of 67 Chinese Usher syndrome probands: high rate of ethnicity specific mutations in Chinese USH patients
Source: Orphanet J Rare Dis. 2015 Sep 4;10:110. doi: 10.1186/s13023-015-0329-3 (PMC4559966; doi:10.1186/s13023-015-0329-3)
Supplement: Additional file 2: Figure S1. — Both Novel missense alleles identified in MYO7A genes are conserved between human, zebrafish and Drosophila melanogaster. (PPTX 676 kb) [file 13023_2015_329_MOESM2_ESM.pptx]

## Slide 1
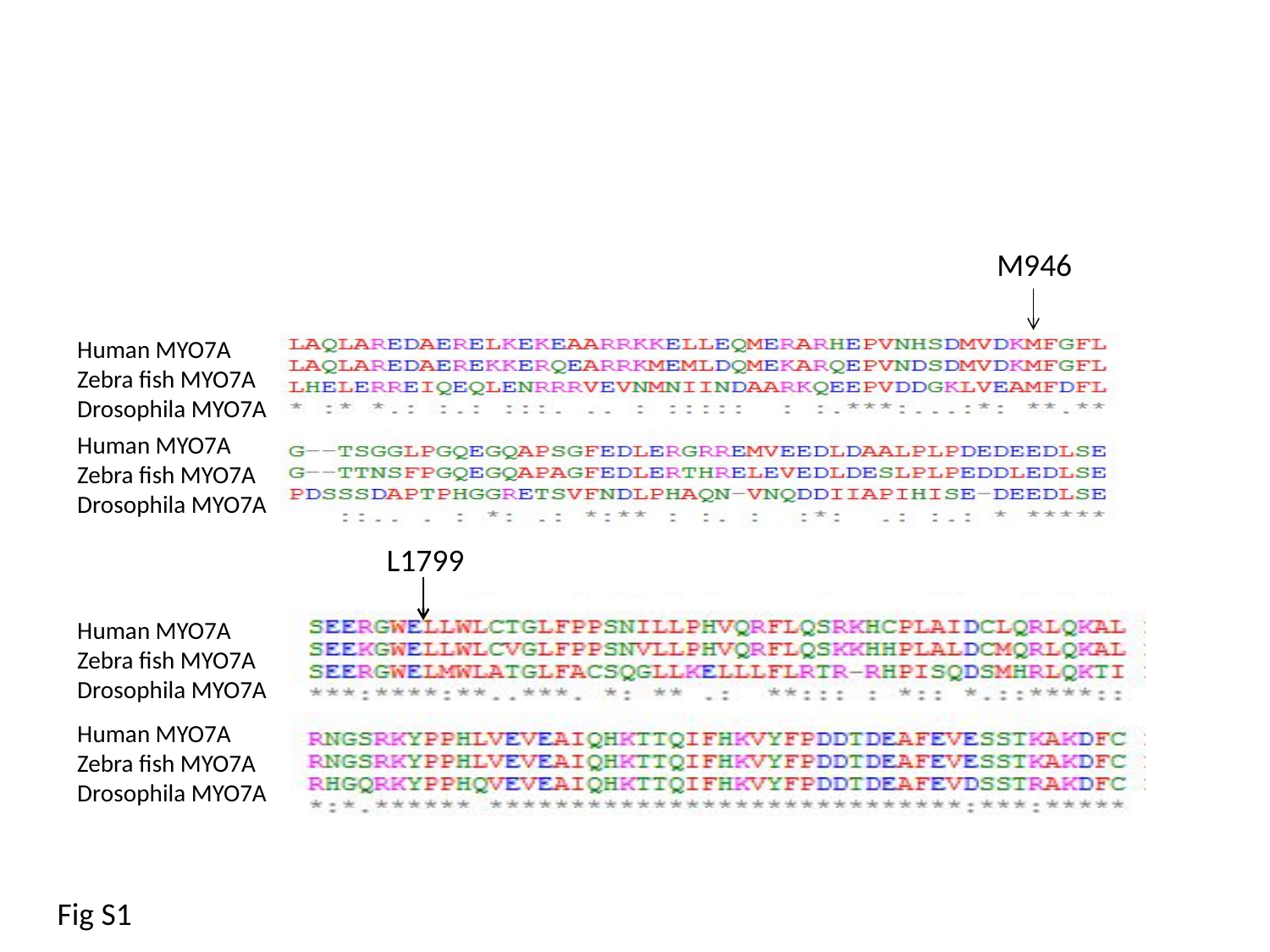

M946
Human MYO7A
Zebra fish MYO7A
Drosophila MYO7A
Human MYO7A
Zebra fish MYO7A
Drosophila MYO7A
L1799
Human MYO7A
Zebra fish MYO7A
Drosophila MYO7A
Human MYO7A
Zebra fish MYO7A
Drosophila MYO7A
Fig S1
